# Supplementary material for: The interactome of intact mitochondria by cross-linking mass spectrometry provides evidence for coexisting respiratory supercomplexes
Source: Mol Cell Proteomics. 2017 Dec 8;17(2):216–32. doi: 10.1074/mcp.RA117.000470 (PMC5795388; doi:10.1074/mcp.RA117.000470)
Supplement: Supplemental Data [file supp_RA117.000470_133922_0_supp_23978_fzffwf.zip › spectra_annotation/mito_spectra_annotation/9-1-24-1-24-1.pdf]

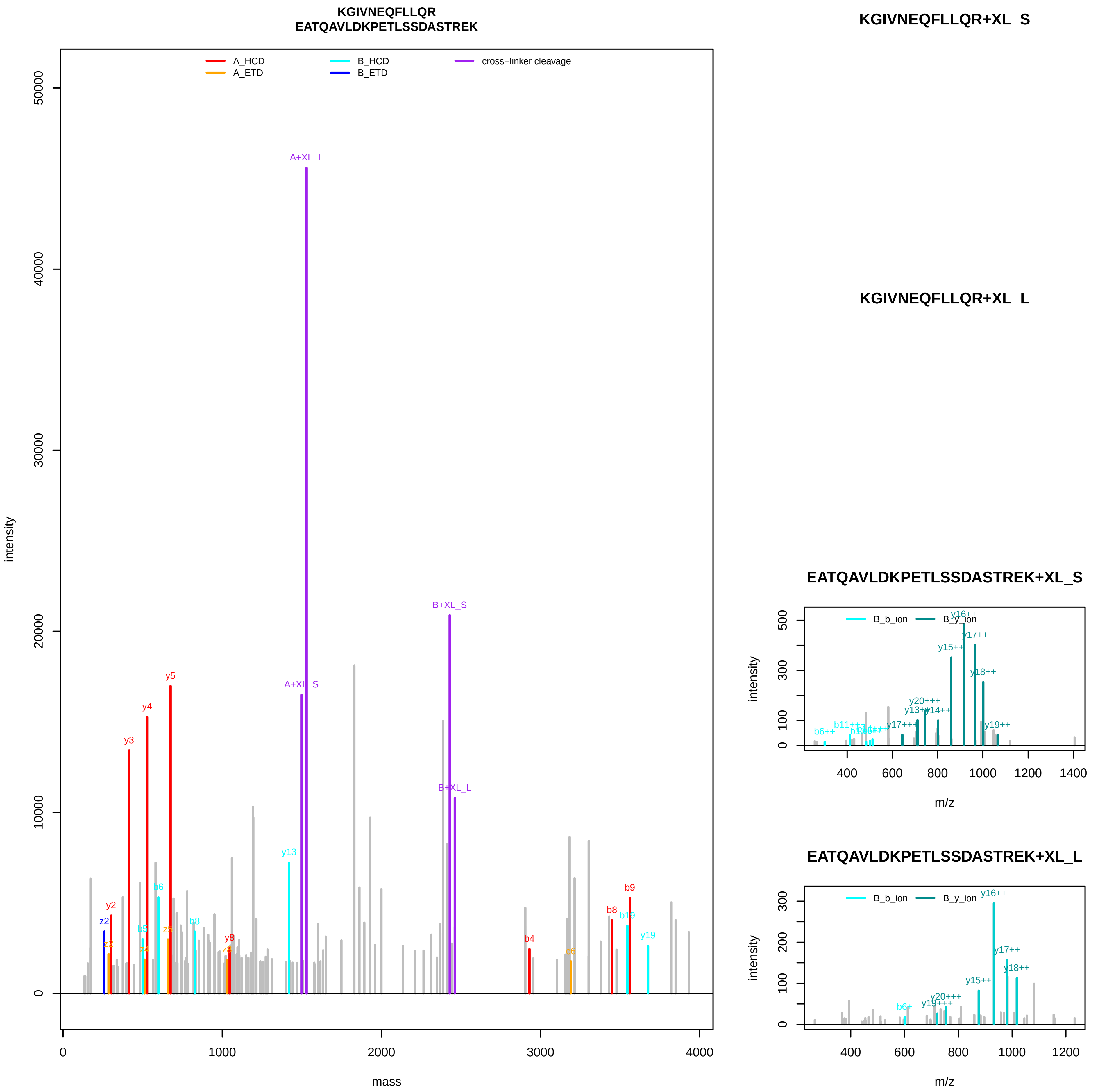
KGIVNEQFLLQR
EATQAVLDKPETLSSDASTREK
A_HCD
A_ETD
B_HCD
B_ETD
cross−linker cleavage
A+XL_L
A+XL_S
B+XL_S
B+XL_L
y2
y3
y4
y5
y8
y13
y19
z2
b4
b5
b6
b8
b9
b19
intensity
mass
0
10000
20000
30000
40000
50000
1000
2000
3000
4000
KGIVNEQFLLQR+XL_S
KGIVNEQFLLQR+XL_L
EATQAVLDKPETLSSDASTREK+XL_S
B_b_ion
B_y_ion
y16++
y17++
y15++
y18++
y20+++
y19++
b6++
b11+++
m/z
EATQAVLDKPETLSSDASTREK+XL_L
y19+++
b6+
